# A Reduction in Mitophagy Is Associated with Glaucomatous Neurodegeneration in Rodent Models of Glaucoma

**DOI:** 10.3390/ijms252313040

**Published:** 2024-12-04

**Authors:** Renuka M. Chaphalkar, Bindu Kodati, Prabhavathi Maddineni, Shaoqing He, Calvin D. Brooks, Dorota L. Stankowska, Shaohua Yang, Gulab Zode, Raghu R. Krishnamoorthy

**Affiliations:** 1 Department of Pharmacology and Neuroscience, University of North Texas Health Science Center, Fort Worth, TX 76107, USA; renukachaphalkar@my.unthsc.edu (R.M.C.); bindu.kodati@unthsc.edu (B.K.); calvinbrooks@my.unthsc.edu (C.D.B.); shaohua.yang@unthsc.edu (S.Y.); 2 North Texas Eye Research Institute, University of North Texas Health Science Center, Fort Worth, TX 76107, USA; dorota.stankowska@unthsc.edu; 3 Department of Ophthalmology, School of Medicine, University of Missouri, Columbia, MO 65212, USA; pmbnv@health.missouri.edu; 4 Department of Pathology, Children’s Health at Dallas, Dallas, TX 75235, USA; shaoqinghe@gmail.com; 5 Department of Microbiology, Immunology and Genetics, College of Biomedical and Translational Sciences at University of North Texas Health Science Center, Fort Worth, TX 76107, USA; 6 Center for Translational Vision Research, Gavin Herbert Eye Institute, University of California, Irvine, CA 92697, USA; gzode@hs.uci.edu

**Keywords:** glaucoma, neurodegeneration, mitophagy

## Abstract

Glaucoma is a heterogenous group of optic neuropathies characterized by the degeneration of optic nerve axons and the progressive loss of retinal ganglion cells (RGCs), which could ultimately lead to vision loss. Elevated intraocular pressure (IOP) is a major risk factor in the development of glaucoma, and reducing IOP remains the main therapeutic strategy. Endothelin-1 (ET-1), a potent vasoactive peptide, has been shown to produce neurodegenerative effects in animal models of glaucoma. However, the detailed mechanisms underlying ET-1-mediated neurodegeneration in glaucoma are not completely understood. In the current study, using a Seahorse Mitostress assay, we report that ET-1 treatment for 4 h and 24 h time points causes a significant decline in various parameters of mitochondrial function, including ATP production, maximal respiration, and spare respiratory capacity in cultured RGCs. This compromise in mitochondrial function could trigger activation of mitophagy as a quality control mechanism to restore RGC health. Contrary to our expectation, we observed a decrease in mitophagy following ET-1 treatment for 24 h in cultured RGCs. Using Morrison’s model of ocular hypertension in rats, we investigated here, for the first time, changes in mitophagosome formation by analyzing the co-localization of LC-3B and TOM20 in RGCs. We also injected ET-1 (24 h) into transgenic GFP-LC3 mice to analyze the formation of mitophagosomes in vivo. In Morrison’s model of ocular hypertension, as well as in ET-1 injected GFP-LC3 mice, we found a decrease in co-localization of LC3 and TOM20, indicating reduced mitophagy. Taken together, these results demonstrate that both ocular hypertension and ET-1 administration in rats and mice lead to reduced mitophagy, thus predisposing RGCs to neurodegeneration.

## 1. Introduction

Glaucoma is an optic neuropathy characterized by the progressive degeneration of the optic nerve and the loss of retinal ganglion cells (RGCs) leading to visual impairment. Primary open angle glaucoma (POAG), the most common form of the disease, is typically associated with elevated intraocular pressure (IOP) [1,2]. IOP is the only treatable risk factor in glaucoma, and all current therapies for glaucoma are aimed at lowering IOP. IOP-lowering approaches remain the gold standard for glaucoma therapy; however, they do not completely prevent progression of RGC loss [3,4]. Hence, neuroprotective therapies are sorely needed in addition to IOP-lowering modalities to delay axonal injury and enhance the survival of RGCs for the clinical management of glaucoma.

Multiple factors have been shown to contribute to glaucomatous neurodegeneration, including oxidative stress [5,6], glutamate excitotoxicity [7], release of tumor necrosis factor (TNF-α) [8,9], and mitochondrial dysfunction [10,11,12]. One of the key factors implicated in the pathogenesis of glaucoma is endothelin-1 (ET-1), a 21-amino-acid vasoactive peptide belonging to the endothelin family [13,14,15]. Previous studies reported that ET-1 levels are elevated in the aqueous humor and circulation of patients with POAG [16,17,18] and in animal models of glaucoma [19,20]. Peribulbar and intravitreal ET-1 administration has been shown to produce degeneration of optic nerve axons and RGC loss [13,21,22,23,24,25]. ET-1 acts through two classes of G-protein coupled receptors: endothelin receptor A (ET_A_) and endothelin receptor B (ET_B_). Both ET_A_ and ET_B_ receptors are abundantly expressed in various ocular tissues, including retinal ganglion cells in the retina [14,26,27]. An increase in ET_B_ receptor expression was observed following IOP elevation in Brown Norway rats, and neurodegenerative changes were significantly attenuated in ET_B_ receptor knockout rats compared to wild type rats, which is indicative of a causative role of the ET_B_ receptor in glaucomatous neurodegeneration [14]. However, the detailed mechanisms underlying ET-1-mediated RGC loss in glaucoma are still not clearly understood.

We have shown recently that ET-1 treatment in cultured RGCs resulted in a significant decline in ATP synthase expression, leading to impaired mitochondrial bioenergetics, which could be one mechanism underlying the ET-1-mediated neurodegeneration of RGCs in glaucoma [11]. We hypothesize that this decline in mitochondrial function in RGCs may lead to mitochondrial damage, which in turn would require activation of the mitophagy pathway as a quality control mechanism to maintain cellular viability. Mitochondria are the major source of adenosine triphosphate (ATP) synthesis and are essential for neuronal survival and the propagation of axon potentials. Neurons are highly dependent on mitochondrial respiration to maintain synaptic transmission and axonal transport, which are crucial for the survival and functioning of the RGCs. Thus, mitochondrial dysfunction could be detrimental to neuronal health and function. Dysfunctional mitochondria are normally trafficked to the lysosome for degradation via mitophagy, and defects in this pathway have been implicated in many neurodegenerative diseases [28,29]. Mitophagy is a highly specialized form of autophagy. It involves the selective removal of damaged mitochondria in response to various stress conditions, including increased production of reactive oxygen species (ROS) [30], hypoxia [31], mitochondrial depolarization [32], and protein misfolding. Hence, mitophagy plays a pivotal role in preventing the accumulation of dysfunctional mitochondria, thereby possibly protecting against neuronal death.

Dysregulation of mitophagy (either an increase or a decrease) contributes to the pathology of several neurodegenerative diseases, including glaucoma [33,34,35,36]. The present study was aimed at evaluating the effects of endothelin treatment on mitochondrial function and mitophagy in RGCs and in an ocular hypertension model of glaucoma. In this study, we observed a decrease in the mitophagosome density in cultured RGCs following ET-1 treatment for 24 h, and we found a similar decline in mitophagosomes in RGCs after two weeks of IOP elevation in adult Brown Norway rats. Our data suggest that ET-1 treatment causes a decline in mitophagy, with the potential implication of an accumulation of defective mitochondria, which may contribute to RGC death in a rodent model of glaucoma.

## 2. Results

### 2.1. ET-1 Significantly Decreases Mitochondrial Respiration Following ET-1 Treatment in Primary RGCs

Our previous data showed that ET-1 treatment for 4 h and 24 h significantly decreased the rate of ATP production in cultured RGCs [11]. This indicated the possibility that ET-1 treatment generated energy deficits in the primary RGCs, which prompted us to evaluate the effect of ET-1 on mitochondrial respiration. We determined the oxygen consumption rate (OCR) following ET-1 treatment for 4 h and 24 h using the Seahorse XF Cell Mito Stress Test kit (Agilent Technologies, Inc., Santa Clara, CA, USA), before and after injecting oligomycin, carbonylcyanide-p-trifluoromethoxyphenylhydrazone (FCCP), and rotenone/antimycin A. The OCR measurements enabled us to measure the key parameters of mitochondrial function, including basal respiration, maximal respiration, ATP-linked mitochondrial respiration, proton leak, and spare respiratory capacity.

**Figure 1:**
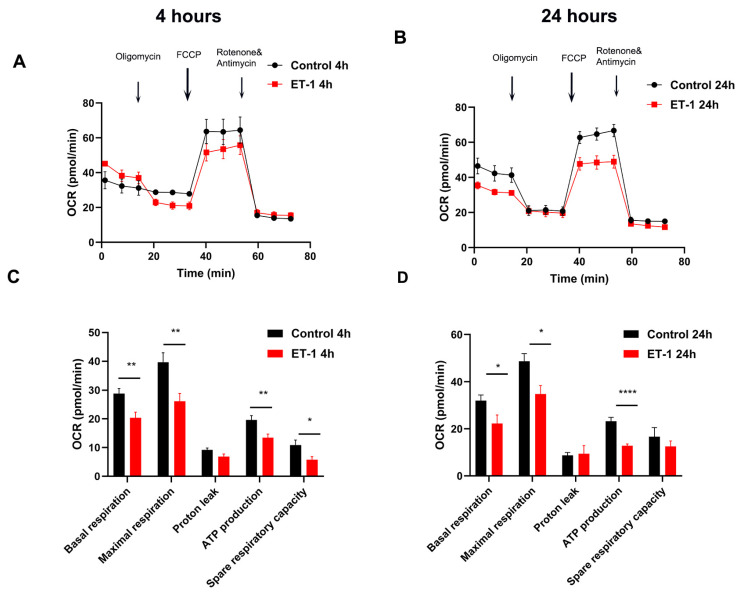
ET-1 decreases oxygen consumption rate (OCR) at 4 h and 24 h in primary RGCs. (**A**). Representative OCR profiles showing OCR recordings at baseline and after treatment with oligomycin, FCCP, and rotenone/Antimycin A following ET-1 treatment for 4 h. (**B**). Representative OCR profiles showing OCR recordings at baseline and after treatment with oligomycin, FCCP, and rotenone/Antimycin A following ET-1 treatment for 24 h. (**C**). Bar graphs showing quantitation of oxygen consumption rate during basal respiration, maximal respiration, ATP-linked respiration, spare respiratory capacity, and proton leak following ET-1 treatment for 4 h. (**D**). Bar graphs showing quantitation of oxygen consumption rate during basal respiration, maximal respiration, ATP-linked respiration, spare respiratory capacity, and proton leak following ET-1 treatment for 24 h. Data represented as the mean ± SEM, (Student’s *t*-test, n = 3 biological replicates per group), significance at * *p* < 0.05, ** *p* < 0.01, **** *p* < 0.0001.

Representative OCR profiles in primary RGCs following ET-1 treatment for 4 h and 24 h are depicted in Figure 1A,B. After oligomycin injection, the OCR was decreased for the 4 h and 24 h time points of ET-1 treatment (Figure 1A,B). At the 4 h time point of ET-1 treatment, we observed a significant decrease of 29% in the basal rate of respiration from 28.8 ± 1.7 to 20.38 ± 1.9 (*p* = 0.006) (Figure 1C). This decrease indicates a significant decline in the endogenous ATP synthetic capacity of the cells undergoing ET-1 treatment. The maximal respiration was significantly reduced by 34%, with a decrease from 39.7 ± 3.2 to 26.2 ± 2.7 (*p* = 0.008) (Figure 1C). A decrease in maximal respiratory capacity is revealed by a reduction in the FCCP-stimulated respiration of the cells, since the protonophore FCCP dissipates the proton gradient and causes the mitochondria to function at maximal activity, resulting in rapid oxidation of mitochondrial substrates to meet this metabolic challenge. This significant decrease in the maximal respiratory OCR of the ET-1-treated RGCs compared to the control indicates the effect of ET-1 to cause mitochondrial dysfunction (a decreased capacity to respond to an increased energy demand). There was also a significant 31% decrease in ATP-linked OCR following ET-1 treatment compared to control from 19.6 ± 1.5 to 13.50 ± 1.2 pmol/min (*p* = 0.007) (Figure 1C). The spare respiratory capacity, a measurement of the difference between basal respiration and protonophore-stimulated respiration, was significantly reduced at 4 h by 53% in ET-1-treated cells compared to the control. The spare respiratory capacity was decreased from 10.8 ± 1.7 to 5.8 ± 1.0 pmol/min (*p* = 0.036) with ET-1 treatment for 4 h (Figure 1C). This signifies a decreased capability of the RGCs to respond to an increased demand of ATP and withstand metabolic stress within the cells.

For the 24 h time point of ET-1 treatment, there was a decrease in basal respiration with ET-1 by 30% from 32.0 ± 2.4 to 22.3 ±3.5 pmol/min (*p* = 0.03) compared to the control RGCs (Figure 1D). There was a significant 28% decrease in maximal respiration from 48.6 ± 3.2 to 34.8 ± 3.5 pmol/min (*p* = 0.01) (Figure 1D). ATP-linked respiration was reduced by 45% from 23.2 ± 1.6 to 12.8 ± 0.7 pmol/min (*p* < 0.0001) (Figure 1D). There was an appreciable decrease of 25% in the spare respiratory capacity from 16.7 ± 3.8 to 12.5 ± 2.3, although it was not statistically significant.

In addition, the decline in ATP-linked OCR observed with ET-1 at 24 h was more drastic than at 4 h. No significant difference was observed in proton leak at both time points compared to the control (n = 3 biological replicates).

### 2.2. ET-1 Increases Reactive Oxygen Species in Primary RGCs

**Figure 2:**
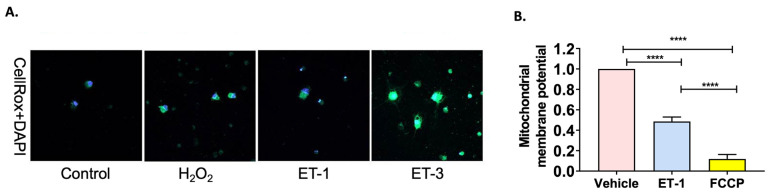
Endothelin treatment elevates reactive oxygen species in cultured primary RGCs. (**A**). Primary RGCs were either untreated (control), or treated with H_2_O_2_ (positive control), ET-1, or ET-3 for 1 h. Cells were stained with CellRox (Green) to detect reactive oxygen species and nuclear dye DAPI (Blue). (**B**). Mitochondrial membrane potential determined by JC-1 assay in RGCs treated with vehicle or ET-1 for 4 h. FCCP (100 μM), an uncoupler of oxidative phosphorylation, was used as positive control. Experiments were performed in triplicate. Data are represented as mean  ± SEM (**** *p* < 0.0001) (one-way ANOVA followed by Tukey’s multiple comparisons test).

Many studies indicate that oxidative stress is an important factor that contributes to neurodegeneration in animal models of glaucoma. To assess the effect of ET-1 and ET-3 treatment on the generation of oxidative stress, we measured reactive oxygen species using the CellROX Green reagent in primary RGCs following the endothelin treatments. As a positive control, primary RGCs were treated with hydrogen peroxide (which produces a robust increase in ROS), which showed and was found to generate intense staining with the CellROX reagent, demonstrating the effective detection of reactive oxygen species using the assay reagent, thereby authenticating the assay reagents and pointing to the veracity of the findings. Primary RGCs treated with ET-1 for 1 h produced more ROS compared to control primary RGCs (Figure 2A). The ET_B_ receptor agonist ET-3 produced a more pronounced increase in ROS, indicated by a drastic increase in fluorescence after treatment with CellROX.

### 2.3. ET-1 Decreases Mitochondrial Membrane Potential in Primary RGCs

To assess changes in the mitochondrial membrane as potential indicatiosn of mitochondrial health, we treated RGCs with the JC-1 dye. Primary RGCs were treated with either ET-1, vehicle, or FCCP (positive control) for 4 h. The intensity of the fluorescence signal was measured by Cytation 5 (BioTekInc., Winooski, VT, USA). A reduction in the ratio of Red/Green is indicative of a decrease in mitochondrial potential and indicative of mitochondrial membrane damage. FCCP (100 µM), a protonophore, served as a positive control for the JC-1 dye/mitochondria depolarization. Following ET-1 treatment for 4 h, we found a significant decline in the mitochondrial membrane potential from 1.0 ± 1.6 to 0.49 ± 0.04 (*p* < 0.0001) compared to the control-treated primary RGCs (Figure 2B). As expected, treatment with the protonophore produced a complete decline of the mitochondrial potential (0.12 ± 0.04, *p* < 0.0001) which was reflected in the diminished red to green fluorescence ratio.

### 2.4. ET-1 Decreases Co-Localization of Mitotracker and Lysotracker in Primary RGCs

An essential step in the mitophagy pathway is the recruitment of damaged mitochondria to the autophagosomes, a double-membraned structure that incorporates the Atg family protein, LC3, which is a selective autophagosome marker. We investigated whether there is a change in the mitophagy with ET-1 compared to the basal levels (control) in primary RGCs.

**Figure 3:**
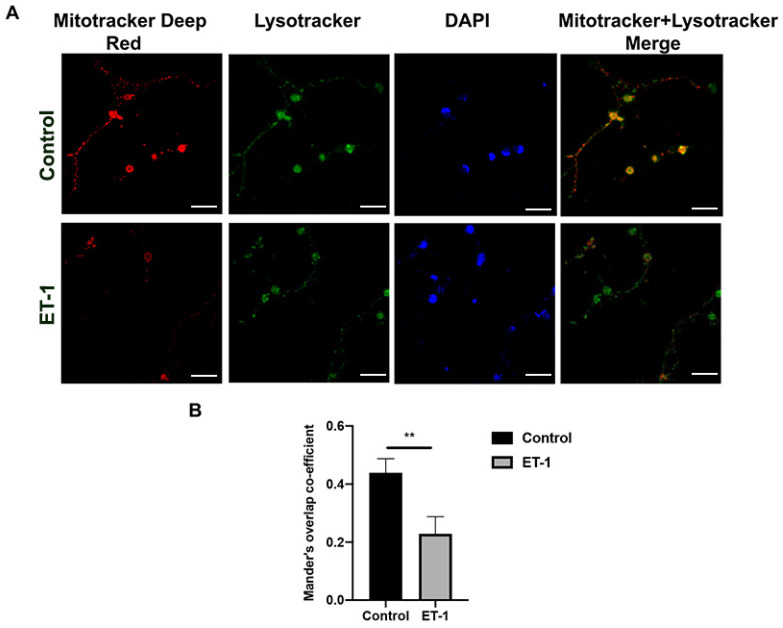
ET-1 treatment mediated decrease in co-localization of Lysotracker (Green) and Mitotracker (Red) in cultured primary RGCs were stained with Mitotracker Deep Red, Lysotracker Red and nuclear dye DAPI (Blue) following ET-1 treatment for 24 h. (**A**). A decrease in co-staining (yellow) of mitotracker and lysotracker was found following ET-1 treatment indicative of decreased mitophagy. (**B**). Quantitation of co-localization puncta was determined by Mander’s overlap co-efficient. Scale bar = 20 µm. Data represented as the mean ± SEM, (Student’s *t*-test, n = 3 biological replicates per group), significance at ** *p* < 0.01.

The prominent co-staining of MitoTracker red and Lysotracker in control RGCs is suggestive of robust mitophagy occurring under basal conditions (Figure 3A). However, following a 24 h ET-1 treatment, there was a decrease in the co-localization of MitoTracker and LysoTracker fluorescence, suggesting a reduction in mitophagy compared to the untreated control cells. The merge panel showed that, with ET-1 treatment, there was a decrease in co-localization puncta of MitoTracker and LysoTracker. Quantitation of the co-localization pixels between MitoTracker and LysoTracker was carried out by measuring the Mander’s overlap co-efficient (MOC) using a Coloc2 plugin on ImageJ. There was a significant decrease in MOC values from 0.43 ± 0.04 to 0.22 ± 0.04 (*p* < 0.01, n = 3 per group, Student’s *t*-test) (Figure 3B). These results indicate a significant decrease in mitophagy in primary RGCs with ET-1 treatment (24 h) compared to the control.

### 2.5. ET-1 Treatment for 24 h Decreases Autophagosome Formation in GFP-LC3 Transgenic Mice in Retinal Ganglion Cells

**Figure 4:**
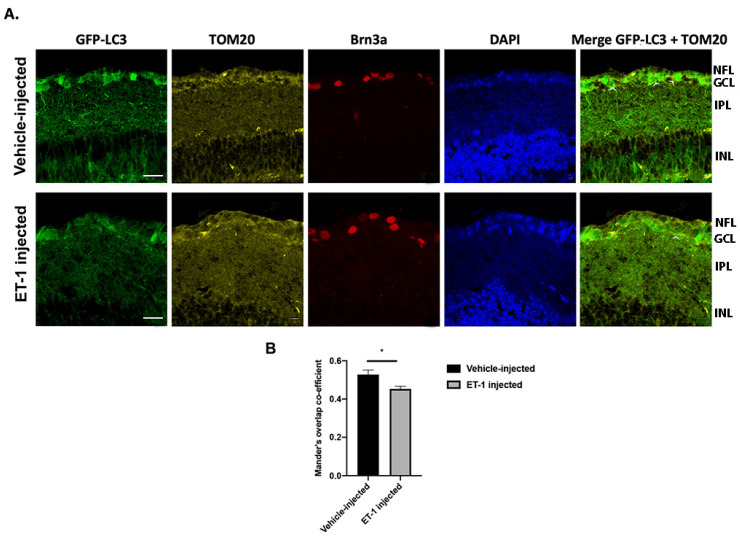
Intravitreal ET-1 administration in GFP-LC3 transgenic mice decreased autophagosome formation in retinal ganglion cells. (**A**). OCT sections showing a significant decrease in co-localization of GFP-LC3 (green) with TOM20 (yellow) 24 h following intravitreal ET-1 injection (white arrow heads indicate the co-localization of GFP-LC3 and TOM20 in GCL layer). Brn3a immunostaining (red) was used to detect RGCs and additional staining was done with nuclear dye DAPI (Blue). (**B**). Quantitation of co-localization of GFP-LC3 with TOM20 determined by Mander’s overlap co-efficient (n = 3, * *p* < 0.05). Scale bar =20 µm. Data represented as mean ± SEM. NFL: nerve fiber layer, GCL: ganglion cell layer, IPL: inner plexiform layer, INL: inner nuclear layer.

To determine if ET-1 mediates these changes in mitophagosome formation, we carried out in vivo studies using GFP-LC3 transgenic mice. The mice were intravitreally injected with ET-1 (1 nmole) (24 h) in one eye while the contralateral eye was injected with a vehicle (water). Analysis of the co-localization puncta corresponding to LC3B-positive and TOM20-positive overlapping pixels was carried out. Co-localization of LC3B and TOM20 in the retina was higher in the vehicle-treated eyes, compared to the ET-1-injected eyes (Figure 4A). Quantitation analysis was carried out by estimating the Mander’s overlap co-efficient. There was a significant decrease in the tM1 or tM2 values from 0.52 ± 0.02 to 0.45 ± 0.01 (*p* = 0.01, n = 3, Student’s *t*-test) (Figure 4B). These results suggest that ET-1 treatment for 24 h decreases the number of mitophagosomes in the ganglion cell layer of the retina.

### 2.6. Elevated IOP Decreases Formation of Mitophagosomes in Retinal Ganglion Cells of Adult Brown Norway Rats

**Figure 5:**
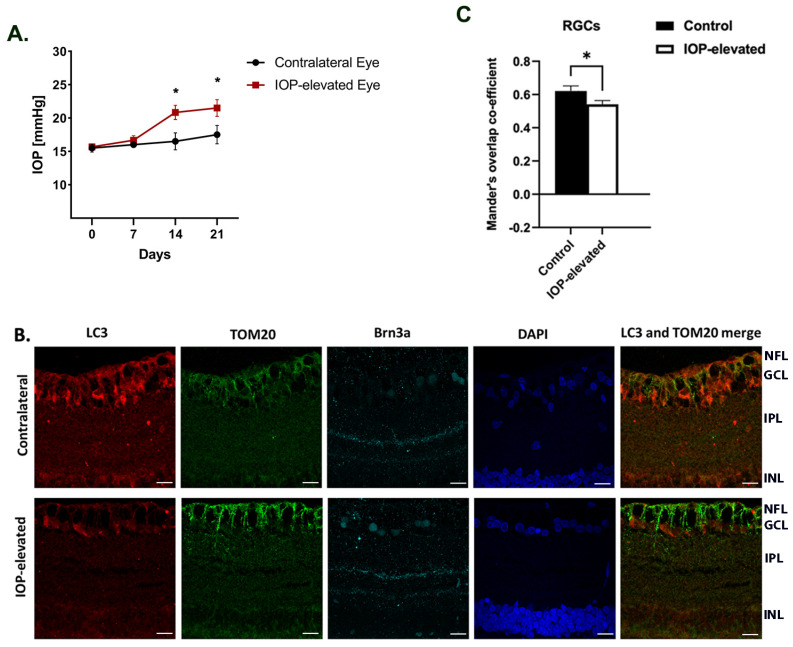
Elevated IOP in Brown Norway rats decreased the formation of mitophagosomes in retinal ganglion cells. (**A**). IOP was elevated in one eye of rats by the Morrison model and maintained for 2 weeks. Representative graph of IOP measurements for IOP elevated (red squares) and contralateral control (black circles) eyes in adult retired breeder Brown Norway rats. (**B**). Retina sections obtained from rat eyes were stained using anti-LC3B (marker of autophagosomes) and anti- TOM20 (outer mitochondrial membrane protein). Brn3a immunostaining (cyan) was used to detect RGCs and additional staining was done with nuclear dye DAPI (blue). Retinas from IOP elevated rat eyes showed a significant decrease in co-localization puncta in RGCs. (**C**). Quantitation of co-localization of LC3B (red) with TOM20 (green) was determined by assessment of Mander’s overlap co-efficient (n = 6, * *p* < 0.001). Scale bar = 20 µm. Data represented as mean  ± SEM. NFL: nerve fiber layer, GCL: ganglion cell layer, IPL: inner plexiform layer, INL: inner nuclear layer.

Injection of hypertonic saline into episcleral veins was used to elevate IOP in one eye of Brown Norway rats [37] while the other eye served as a contralateral control. As seen in Figure 5A, IOP elevation was first detected 7–14 days after surgery and remained elevated for 2 weeks, generating 77 mmHg-Days of IOP exposure (average of n = 6 rats), following which the rats were humanely euthanized, and the eyes collected for paraffin sectioning.

Retina sections were obtained from rats and immunostaining was carried out using the antibodies to LC3B and TOM20. We measured the cellular distribution of the autophagosome marker protein LC3B and the mitochondrial outer membrane marker Tom20, specifically in the RGC layer of these retina sections. The LC3B antibody used for the study recognizes the cytosolic LC3 form, LC3B-I, as well as its autophagosome membrane-bound form, LC3B-II. The number of mitochondrial autophagosomes in the retinas of the IOP-elevated eyes were compared to those in the contralateral eyes by quantifying the co-localization puncta between LC3B and TOM20. Images obtained from the confocal image Z-stacks were analyzed to estimate the Mander’s overlap co-efficient in the ganglion cell layer. The co-localization puncta were significantly decreased in IOP-elevated eyes compared to the contralateral eye (Figure 5B). IOP elevation significantly decreased the tM value 0.62 ± 0.03 to 0.51 ± 0.03 (*p* < 0.01, n = 6 retinas, Student’s *t*-test) (Figure 5C).

The data indicated a significant decline in mitophagosome formation in IOP-elevated rat eyes compared to the contralateral eyes. 

## 3. Discussion

Previous studies have reported that mitochondrial dysfunction is implicated in the development of age-related neurodegenerative diseases including Alzheimer’s, Parkinson’s, and Huntington’s diseases. Increasing evidence now suggests that there is an involvement of ROS causing oxidative imbalance and oxidative damage in the pathogenesis of these neurodegenerative disorders [38,39,40]. Several review articles point to the involvement of chronic oxidative stress and accumulation of ROS leading to RGC loss in glaucoma [41,42,43]. However, there is a lack of explanation of the cause of vulnerability of RGCs to mitochondrial damage. One plausible explanation underlying the susceptibility of the RGCs to mitochondrial dysfunction is their high metabolic rate and energy demand. Mitochondrial biosynthesis occurs in the RGC soma located in the ganglion cell layer, but the energy requirements extend to axonal as well as dendritic arbors projecting into the inner plexiform layer to establish synaptic connections with other retinal neurons. Hence, the synapses in dendritic arbors are densely packed with mitochondria because an extraordinarily high amount of energy is required to synthesize and release neurotransmitters, organize the synaptic vesicle pool, regulate calcium homeostasis and restore the ion gradients at the active sites [44].

In this study, we have investigated the effects of ET-1 treatment on mitochondrial function at two time points (4 h and 24 h) in cultured RGCs. Measurement of cellular respiration is a useful technique to assess cellular bioenergetics due to the link between ATP production and oxygen consumption during oxidative phosphorylation [45]. We found that, at both time points of ET-1 treatment, there was a significant decline in the key mitochondrial bioenergetic parameters, including ATP-linked respiration, basal respiration, maximal respiration, and spare respiratory capacity (Figure 1). Excitable cells such as neurons go through periods of high energy demand, hence protonophore-stimulated respiration is a reflection of the maximal respiratory capacity of the cells. The spare respiratory capacity is the difference between basal respiration and protonophore-stimulated respiration and is an indication of the cell’s ability to meet an increased energy demand or withstand periods of stress [45]. Taken together, our findings suggest that one of the mechanisms underlying ET-1-mediated neurodegeneration in RGCs involves mitochondrial dysfunction and alterations in oxidative phosphorylation pathway. In our previous study, we observed a decreasing trend in the expression of key mitochondrial proteins, cytochrome c oxidase copper chaperone (COX17) (3-fold), ATP synthase, and H^+^ transporting mitochondrial F0 complex (ATP5H), following 2 weeks of IOP elevation in Morrison’s model of glaucoma [11]. A decrease in the critical components of the electron transport chain and oxidative phosphorylation machinery could create conditions favorable for the generation of reactive oxygen species. As seen in Figure 2, there was an increase in ROS following the treatment of RGCs with ET-1 and a pronounced elevation of ROS following activation of ET_B_ receptors by treatment with the ET_B_ receptor agonist ET-3. These findings are in alignment with our previous observations of the neurodegenerative effects of the ET_B_ receptor during ocular hypertension in rats [14]. With the induction of ocular hypertension either by chronic or acute methods, the RGCs could have an increase in reactive oxygen species and thereby result in oxidative stress. Accumulation of ROS can produce changes in cellular redox state that, in turn, could produce functional changes in the retina, which include cellular responses and protein-protein and DNA-protein interactions involving transcription factors. Increased oxidative stress by ET_B_ receptor activation could be a key contributor leading to the impairment of mitochondrial bioenergetics in RGCs. For instance, Lau et al., (2006) have demonstrated an appreciable increase in superoxides in the inferior mesenteric ganglion in rats infused intravenously with sarafotoxin (an ET_B_ receptor agonist) [23]. One possible mechanism by which ET_B_ receptor activation elevates oxidative stress is through the activation of NADPH oxidase [46,47]. Reactive oxygen species, including superoxides, are known to induce the opening of the permeability transition pore (created by the juxtaposition of adenine nucleotide translocator and voltage-dependent anion channel), dissipating the mitochondrial membrane potential and thereby producing mitochondrial damage and compromising the ability to synthesize ATP. Therefore, sustaining mitochondrial potential and function is critically important to responding to oxidative stress and maintaining the quality control of mitochondria.

Impairment of mitochondrial bioenergetics in primary RGCs following ET-1 implied the possibility of a compromise in the mitochondrial quality control system. Maintaining a healthy mitochondrial network requires the clearance of defective mitochondria, and one of the mechanisms of mitochondrial clearance occurs via a process called mitophagy [48]. Mitophagy is the principal pathway by which a cell can eliminate damaged mitochondria, which makes it a crucial component of mitochondrial quality control. We observed a decrease in mitophagy in cultured RGCs with 24 h of ET-1 treatment (Figure 3). This process is initiated by sequestering the individual damaged mitochondria by recruiting the phagophore protein LC3, leading to the formation of mitophagosomes and their subsequent fusion with lysosomes [49]. In the present study, we assessed the changes in mitophagosome formation following IOP elevation of 2 weeks in adult Brown Norway rats. Our in vivo study data showed a significant decrease in co-localization of LC3B-TOM20, indicating a decline in mitophagosome formation in the RGC layer (Figure 5). Other investigators have found overexpression of parkin exerted a significant protective effect on RGCs and partially restored dysfunction of mitophagy in response to cumulative IOP elevation [33]. A significant increase in the number of autophagosomes was observed in the optic nerve of aging DBA/2J mice at all ages compared to the age-matched controls, but, surprisingly, these defective mitochondria were not targeted towards autophagosomes indicating lack of mitophagy initiation [50]. These findings suggest impairment of mitophagy contributes to RGC loss during glaucoma. However, other investigators have reported findings to the contrary. Hirt et al., (2018) reported an overactivation of autophagy as a potential cellular mechanism leading to ON degeneration in chronic hypertensive DBA/2J mice [34]. Previous studies have also demonstrated an increase in the number of mitophagosomes and mitophagy observed in glaucomatous D2 mice [51]. Some of these discrepancies in the findings could be due to the glaucoma model and the duration of IOP elevation at which autophagy is being assessed. Further studies are needed to understand these discrepancies.

It is evident from previous publications that differences in experimental models, timing of analysis following the onset of IOP elevation, and the age of the animals are critical in evaluation of activation of autophagy and measurement of autophagic flux. In 12-month-old DBA/2J mice, there was a decrease in protein levels of LC3, p62, and LAMP1 in RGCs, suggesting a decreased autophagy, which is contradictory to several other studies. However, in RGCs of DBA/2J::GFP-LC3 mice, an increased amount of autophagosome-associated LC3 (LC3-II, LC3 puncta) was found; however, the autophagic flux was diminished in the outflow pathway of DBA/2J hypertensive mice compared to age-matched C57BL/6J [34]. Meanwhile, our LC3-TOM20 co-localization data in GFP-LC3 mice intravitreally injected with ET-1 showed a decrease in the co-localization puncta, indicating a decrease in mitophagosome formation (Figure 4). It can be inferred from all these studies that an increase in autophagic vacuoles or mitophagosomes is not necessarily indicative of an increase in mitophagy or autophagy.

In summary, we have described here for the first time an impairment in the formation of mitophagosomes in RGCs in Morrison’s model of IOP elevation in rats as well as in ET-1-injected GFP-LC3 mice. However, it is unclear how ET-1 mediates cellular changes contributing to a decline in mitophagy and this will be the subject of future investigations. Increased reactive oxygen species can impair several cellular pathways and could be a contributor to the decline in mitophagy we report in the current study. However, we do not have sufficient evidence to rule out ET-1 directly impairing mitophagy. Mitophagy in RGCs has not been studied extensively and it may be advantageous to investigate the mitophagy pathway further, exploring both the initiation of mitophagosome formation and the degradation of mitophagosomes. Whether the mitophagosomes are transported to the lysosomes for degradation, triggering induction of mitophagy, or if there is an accumulation and inefficient fusion of mitophagosomes with lysosomes resulting in a potential defect in the mitophagy pathway remains to be seen. The interplay of different events, including mitochondrial fusion/fission dynamics, mitochondrial biogenesis, and degradation, play a key role in mitophagosome formation and ultimately regulation of the process of mitophagy. Regardless, our findings suggest an impairment in formation of mitophagosomes and mitochondrial dysregulation specifically in RGCs, which may help to understand the mechanism of mitochondrial dysfunction in the neurodegeneration of glaucoma.

## 4. Materials and Methods

### 4.1. Animals

All protocols and procedures were in accordance with the policies of the Association for Research in Vision and Ophthalmology (ARVO) for the use of animals in research and approved by the Institutional Animal Care and Use Committee (IACUC) (animal protocol # IACUC-2020-0029, dated 14 July 2020) at the University of North Texas Health Science Center at Fort Worth, TX, USA. Retired breeder Brown Norway rats (Charles River Laboratories, Wilmington, MA, USA) in the age group of 8 to 12 months were used for elevating ocular hypertension. Homozygous GFP-LC3 (Tg-GFP-LC3/6J) mice, 4 to 5 months old, on a C57BL6 background were a kind gift from Dr. Noboru Mizushima and used to monitor the formation of autophagosomes in vivo [52]. The GFP-LC3 mice were used to quantify the autophagosomes in retina sections obtained following ET-1 intravitreal injection. For the ocular hypertension model, retired breeder Brown Norway rats obtained from Charles River Laboratories (Wilmington, MA, USA) were housed under constant dim lighting (90 lux) to minimize diurnal variations in IOP. All the other animals were housed in 12 h light/12 h dark conditions with a comfortable temperature setting (21 °C to 26 °C) and humidity (40% to 70%). Food and water were provided *ad libitum*.

### 4.2. Evaluation of Mitochondrial Bioenergetics and Mitophagy in Primary Rat RGCs

#### 4.2.1. Primary RGC Isolation

Female timed-pregnant Sprague Dawley rats were obtained from Charles River Laboratories (Wilmington, MA, USA). Primary rat RGCs were isolated using a Thy-1.1 antibody-panning method [53]. Briefly, post-natal day 4-6 rat pups were euthanized, and the isolated retinas were treated with 4.5 units/ mL of papain solution to dissociate the retinal cells. The cell suspension was subsequently incubated for 10 minutes with a rabbit anti-macrophage antibody and transferred to a Petri dish coated with a goat anti-rabbit IgG (H+L chain) antibody for 35 minutes. Cells that were not attached to the coated goat anti-rabbit IgG were transferred to a Petri dish coated with anti-Thy1.1 antibody derived from a T11D7 Hybridoma cell line for 60 min with intermittent shaking. After washing 3 to 5 times with DPBS, RGCs were then dissociated by trypsin treatment and then collected by centrifugation. Isolated RGCs were seeded either directly in a Seahorse XF96 Cell Culture Microplate or on 35 mm Mattek glass bottom dish containing No. 1.5 coverslips, coated with poly-D lysine and mouse-laminin. Cells were cultured in serum-free Dulbecco’s modified Eagle’s medium containing brain-derived neurotrophic factor (BDNF) (50 ng/mL; Peprotech, Rocky Hill, NJ, USA), ciliary neurotrophic factor (10 ng/mL; Peprotech), and forskolin (5 ng/mL; Sigma-Aldrich Corp, St. Louis, MO, USA). Primary RGCs were maintained for a week at 37 °C in a humidified atmosphere of 10% CO_2_ and 90% air to firmly attach and allow neurite outgrowth to occur. One-third volume of the culture medium was changed every two days. The purity of the culture obtained was routinely found to be between 90 and 95% [11]. RGCs from each litter of pups were pooled and represent one biological replicate.

#### 4.2.2. ET-1 Treatment

ET-1 peptide was purchased from Bachem (Torrance, CA, USA) and dissolved in water to obtain the working stock concentrations of 500 μM for intravitreal injections, or 100 μM for cell culture. The seeding density of the primary RGCs was approximately 25,000 cells/well for a Seahorse XF96 Cell Culture Microplate and 20,000 cells/well for a 35 mm Mattek glass bottom dish containing No 1.5 coverslips (0.16–0.19 mm thickness). Primary RGCs were allowed to attach and grow in culture for one week to promote neurite outgrowth and then treated with 100 nM ET-1 (final concentration) for either 4 h or 24 h, while untreated RGCs served as controls.

#### 4.2.3. Seahorse Extracellular Flux Analysis

Agilent Seahorse XFe96 analyzer was used to measure oxygen consumption rate (OCR) and extracellular acidification rate (ECAR). These parameters are assessed under basal respiration, maximal respiration, ATP-linked respiration, spare respiratory capacity and H^+^ (Proton) leak, allowing for a complete view of mitochondrial function. 

The cultured primary RGCs were either untreated or treated with ET-1 for 4 h or 24 h in the trophic factor-free medium. A Seahorse Mito Stress kit was used to determine the effects of ET-1 on mitochondrial respiration in primary RGCs. A day prior to the experiment, the Seahorse sensor cartridge was hydrated with water and incubated in a non-CO_2_ incubator at 37 °C. Two hours before the experiment, the water was replaced with a Seahorse calibrant. One hour before the experiment, an assay medium was prepared as follows: Seahorse XF base medium was supplemented with 1 mM pyruvate, 2 mM glutamine, and 10 mM glucose (Agilent, Santa Clara, CA, USA) and the pH was adjusted to 7.4. The RGC culture medium was then replaced with 180  µL of the supplemented Seahorse XF base medium and the cells were incubated in a non-CO_2_ incubator at 37 °C for 1 h. While the cell culture microplates were incubating for an hour, solutions of oligomycin, FCCP, and Rotenone/Antimycin A (Agilent, Santa Clara, CA, USA) were prepared in the seahorse medium to achieve final concentrations of 1.5 μM, 2 μM, and 0.5 μM, respectively, when injected. The appropriate volumes: 20, 22, and 25 µL of Oligomycin, FCCP, and Rotenone/Antimycin A, respectively, were injected into the drug delivery ports A, B, and C of the hydrated sensor cartridge and loaded into the seahorse XF analyzer to calibrate for 30 min. The calibration plate was replaced with the cell culture plate and oxygen consumption (OCR) and extracellular acidification rate (ECAR) were monitored following the sequential injection of Oligomycin, FCCP, and Rotenone/Antimycin A, with each cycle set as 3 min (mix), 2 min (delay), and measurements for 3 min. The Mito Stress test was conducted at two time points following the ET-1 treatment (4 h and 24 h). Data were normalized to the cell number of each well by using a Calcein AM assay.

#### 4.2.4. Oxidative Stress (Reactive Oxygen Species)

Primary RGCs were either untreated (control) or treated with either H_2_O_2_ (positive control), ET-1 (100 nM) or ET-3 (100 nM), for 1 h. A staining assay using the fluorescent dye CellROX Green (ThermoFisher # C10444) was used. CellROX Green is a fluorogenic probe that measures oxidative stress in live cells to detect reactive oxygen species. CellROX is a cell permeant dye that exhibits weak fluorescence in the reducing environment of the cell; however, upon oxidation, it shifts to a bright green photostable fluorescence (Absorption/Emission maxima: 485/520 nm) and subsequently binds to DNA. Imaging was performed immediately after primary RGCs were stained with CellRox for 20 min.

#### 4.2.5. Mitochondrial Membrane Potential

Mitochondrial membrane potential was measured in primary rat RGCs by using a fluorescence tetraethylbenzimidazolylcarbocyanineiodide (JC-1) assay kit (Abcam, Cambridge, MA, USA). Purified RGCs were seeded in a 96-well clear flat –bottom plate with black wall at density of 10,000 cells/well. Cells treated with/without ET-1 for 4 h were followed with the treatment of JC-1 dye (1μM) for 30 min. The JC-1 dye exhibits potential-dependent accumulation in the mitochondria, indicated by a fluorescence emission shift from green (~529 nm) to red (~590 nm). Alteration in the ionic equilibrium results in mitochondria depolarization and is indicated by a decrease in the red/green fluorescent ratio. Data are represented as the red/green fluorescent ratio.

#### 4.2.6. Assessment of Mitophagy Using Lysotracker and Mitotracker

Primary RGCs were labeled with MitoTracker™ Deep Red FM (ThermoFisher Scientific # M22426, Waltham, MA, USA) and LysoTracker™ Red DND-99 (ThermoFisher Scientific # L7528, Waltham, MA, USA) to stain the mitochondria and lysosomes, respectively, according to manufacturer’s instructions. Colocalization of labeled mitochondria and autophagosomes/lysosomes is a common technique for evaluating mitophagy [36]. Primary RGCs were treated with ET-1 for 24 h, following which we analyzed the co-localization of MitoTracker Deep Red and Lysotracker. In brief, the RGC medium was removed, and cells were washed once with DMEM medium. Prewarmed (37 °C) staining solution containing MitoTracker at the final concentration of 25 nM and Lysotracker at the final concentration of 50 nM were added to the live cells and incubated for 30 min at 37 °C. MitoTracker red allows the visualization of active mitochondria, while Lysotracker was used to label the lysosomes. In cell culture studies, co-localization between MitoTracker and LysoTracker or mitochondrial and lysosomal proteins provides a measurement of mitophagy [54]. Quantification of co-localization puncta further acts as a reliable indicator of mitophagy. Cells were counterstained with Hoechst 33,342 to label cell nuclei and Z-stack images were taken at 40× in a Zeiss LSM 510 laser scanning confocal microscope. Quantitation of co-localization puncta was analyzed by measuring Manders’ overlap coefficient and Pearson’s correlation co-efficient.

### 4.3. Evaluation of Mitophagy In Vivo Using Animal Models

#### 4.3.1. Intravitreal Injection of ET-1

ET-1 peptide was purchased from Bachem (Torrance, CA, USA) and dissolved to a stock concentration of 500 μM. ET-1 dosing used in these experiments was based on previous experiments from various studies [23,24,25]. GFP/LC3 mice were intravitreally injected in one eye with 2 μL of 500 μM ET-1 in water while the contralateral eye was injected with 2 μl water. Mice were humanely euthanized 24 h after injection by carbon dioxide (100%) inhalation. Eyes were collected and cryosectioned.

#### 4.3.2. Immunohistochemistry for GFP-LC3 Mice

Eyes from GFP-LC3 were cryosectioned to preserve the fluorescence of the GFP-tagged LC3B. Eyes were enucleated carefully and fixed immediately in 4% paraformaldehyde solution for 16 h at 4 °C. The fixed eyes were washed briefly with 1× PBS for 15 min and the tissue was submersed in sucrose gradient solutions of 10%, 20%, and 30% sucrose for 16 h at 4 °C. Eyes were embedded in OCT compound and cryopreserved in −80 °C until further use. Using a cryostat, OCT-embedded retinal sections of 10 µm thickness were obtained. The retinal sections were washed with 1X PBS. Blocking was carried out using 5% normal donkey serum containing 5% BSA in PBS for 1 h at room temperature followed by primary antibody incubation for overnight at 4 °C. The primary antibodies used were mouse anti-TOM20 (1:100, #WH0009804M1, Millipore Sigma, Darmstadt, Germany), and goat anti-Brn3a (1:200, #sc31984, Santa Cruz Biotechnology, Dallas, TX, USA). Using antibodies specific to each of the Brn3 family (Brn3a, Brn3b, and Brn3c), Xiang et al. (1995) were able to demonstrate immunostaining specifically in the RGCs in a variety of vertebrate retinas. Furthermore, Nadal-Nicolás et al. (2009) show that Brn3a remains a specific RGC marker even during RGC axon injury [55,56].

Secondary antibody incubation was carried out for 1 h at room temperature. Secondary antibodies used were donkey anti-mouse Alexa 546 (1:1000, Invitrogen, Waltham, MA, USA) to detect TOM20, and donkey anti-goat Alexa 647 (1:1000, Invitrogen) to detect Brn3a.

Retinal sections incubated with no primary antibody served as the blank to assess non-specific staining by the secondary antibodies. Sections were mounted using Prolong^®^ Gold antifade reagent with DAPI (P36931, Invitrogen).

#### 4.3.3. Induction of Ocular Hypertension Using the Morrison Model in Adult Brown Norway Rats

IOP elevation was carried out in one eye of retired breeder Brown Norway rats (n = 6) (Charles River Laboratories, Wilmington, MA, USA), in the age group of 8 to 12 months, by injecting hypertonic saline through episcleral veins [37]. Briefly, rats were sedated by inhalation of isoflurane (4% induction and 2.5% maintenance) and anesthesia was ensured by lack of a toe pinch reflex. Following a small conjunctival incision to expose the episcleral veins, hypertonic saline (1.8 M NaCl) was injected into an episcleral vein using a glass needle (TIP01TW1F, World Precision Instruments) at a rate of 309  µL/min for 10 to 20 s. Approximately 50 to 100  μL of hypertonic saline was injected into the episcleral veins, producing scarring of the trabecular meshwork. The contralateral eye served as a corresponding control. IOPs were measured on conscious rats twice weekly using a hand-held TonoLab tonometer (iCare, Vantaa, Finland). Each reading the device gives is the average of 6 individual measurements, and 10 consecutive readings were averaged for each IOP measurement. IOP plots were generated from IOP values obtained from the surgically treated IOP elevated and contralateral control eyes. The IOP exposure in each rat was computed by the integral product of the extent of IOP elevation and the number of days of IOP elevation (expressed as mm Hg-days). Following two weeks of IOP elevation, rats were humanely euthanized by intraperitoneal followed by intracardiac injection of pentobarbital (120 mg/kg body weight) and eyes were collected and paraffin-embedded.

#### 4.3.4. Immunohistochemical Analysis of TOM20 and LC-3B Expression Brown Norway Rats

Following enucleation, a small incision (approximately 4–5 mm) was made just posterior to the limbus and the eye was fixed in 4% paraformaldehyde (PFA) in phosphate-buffered saline (PBS) for 30 min at room temperature on a rotating shaker. After 30 min of incubation, an incision was made along the entire circumference of the limbus to remove the entire anterior segment and the lens. The posterior segment was fixed in 4% PFA for an additional 3 hours. The tissue was rinsed with 70% ethanol and later embedded in paraffin to preserve tissue morphology. Sagittal retinal sections through the optic nerve head (7 μm) were obtained using a rotary microtome, de-paraffinized in xylene, and rehydrated with a graded series of ethanol. Following permeabilization, retinal sections were blocked for 1 h with 5% normal donkey serum containing 5% BSA in PBS at room temperature and incubated with the appropriate primary antibodies overnight at 4 °C. Primary antibodies used in this experiment were mouse anti-TOM20 (1:100, #WH0009804M1, Millipore Sigma), goat anti-Brn3a (1:200, #sc31984, Santa Cruz Biotechnology), and rabbit anti-LC3B (1:100, #L7543, Millipore Sigma). Following the incubation, the sections were washed with 1X PBS and further incubated with the appropriate donkey secondary antibodies conjugated to Alexa-fluorophores (1:1000, Invitrogen) at room temperature for 1–2 h.

#### 4.3.5. Imaging and Analysis

Z-stack images (40X magnification) were taken using a Zeiss LSM 510 Confocal Laser Scanning microscope for Primary RGCs. Colocalization analysis of primary RGCs stained with either MitoTracker/LysoTracker and LC3B/TOM20 were performed using Coloc2 (Fiji’s plugin for co-localization analysis) [36]. Background subtraction was carried out by a Rolling-Ball Background Subtraction of 50 pixels. Primary RGCs were outlined using the freehand drawing tool. Each RGC was taken as an independent region of interest (ROI) for quantification of fluorescence in the cell culture images. Then, each ROI was averaged and divided by the total number of quantified RGCs. For primary RGCs, 40–50 cells per group (control vs. ET-1) were imaged to quantify the co-localization between MitoTracker and LysoTracker.

For the retina sections, Z-stack images (60× magnification) were taken using a Zeiss LSM 880 Confocal microscope. The RGC layer was manually selected using Brn3a as an RGC marker. A freehand region of interest (ROI) tool was used to outline the RGC layer. On the images, for rats and mice, colocalization of TOM20 and LC3B were completed using the procedure explained in the previous section. Mander’s overlap coefficient corresponding to the TOM20 image channel was used to quantify the number of mitochondria co-localizing with LC3 as described by Dunn et al. (2011) [57]. The Coloc2 plugin on ImageJ, was used to determine the Mander’s overlap coefficient, corresponding to the proportion of colocalized pixels (LC3B and TOM20 positive) over TOM20 positive pixels. tM1 or tM2 (thresholded Mander’s coefficient) value corresponding to TOM20 channel was used for quantitation.

### 4.4. Statistical Analysis

GraphPad Prism 8 (La Jolla, CA, USA) was used to perform statistical analysis. Following a positive Kolmogorov–Smirnov normality test, the two experimental groups (control vs. treated) were compared using either Student’s *t*-test or One Way ANOVA followed by post hoc analysis. Statistical significance of the experimental data were described as * *p*  <  0.05; ** *p*  <  0.01, *** *p* < 0.001, **** *p* < 0.0001. Data are presented as mean  ± SEM.

## Data Availability

The data that support the findings of this study are available from the corresponding author upon request.
